# TRACKing tandem repeats: a customizable pipeline for identification and cross-species comparison

**DOI:** 10.1093/bioadv/vbaf066

**Published:** 2025-04-08

**Authors:** Carolina L Adam, Joana Rocha, Peter Sudmant, Rori Rohlfs

**Affiliations:** Institute of Ecology and Evolution, University of Oregon, Eugene, Oregon 97403, United States; Department of Integrative Biology, University of California, Berkeley, Berkeley, CA 94720, United States; Department of Integrative Biology, University of California, Berkeley, Berkeley, CA 94720, United States; Institute of Ecology and Evolution, University of Oregon, Eugene, Oregon 97403, United States; School of Computer and Data Sciences, University of Oregon, Eugene, OR 97403, United States

## Abstract

**Summary:**

TRACK is a user-friendly Snakemake workflow designed to streamline the discovery and comparison of tandem repeats (TRs) across species. TRACK facilitates the cataloging and filtering of TRs based on reference genomes or T2T transcripts, and applies reciprocal LiftOver and sequence alignment methods to identify putative homologous TRs between species. For further analyses, TRACK can be used to genotype TRs and subsequently estimate and plot basic population genetic statistics. By incorporating key functionalities within an integrated workflow, TRACK enhances TR analysis accessibility and reproducibility, while offering flexibility for the user.

**Availability and implementation:**

The TRACK toolkit with step-by-step tutorial is freely available at https://github.com/caroladam/track.

## 1 Introduction

Tandem repeats (TRs) are repetitive genomic sequences characterized by their abundance in genomes, high mutation rates, and presence of multiple alleles within a population (Gymrek 2017), making them a major source of genetic variation (Kashi *et al.* 1997). By accumulating mutations faster than single nucleotide polymorphisms (SNPs) (Sun *et al.* 2012), TRs are an easy target for natural selection and act as central players in rapid evolution (Gemayel *et al.* 2010). Although ubiquitous in all eukaryotic genomes (Richard *et al.* 2008), TRs gained prominence in human and non-human primate studies due to their role as epigenetic and gene expression modulators and their association with many human diseases (Gymrek *et al.* 2016). TR expansions, for instance, are linked to the pathogenesis of multiple types of cancer (Erwin *et al.* 2023) and neurological-related conditions, such as Huntington’s disease (MacDonald *et al.* 1993) and Friedreich’s ataxia (Campuzano *et al.* 1996).

The cumulative evidence that TRs are associated with the evolution of complex traits (Press *et al.* 2014) and likely evolved under selective pressures (Liang *et al.* 2015) highlights the importance of understanding their variation across species through comparative analyses. In great apes, e.g. there is evidence for the role of TRs in chromosomal rearrangements (Farré *et al.* 2011) and gene expression divergence (Bilgin Sonay *et al.* 2015). Thus, building comparative TR frameworks can provide critical insights into evolutionary processes.

The propensity of TRs for homoplasy, coupled with sequencing technology limitations that hindered accurate sequencing of long TRs, has historically restricted their widespread use in cross-species comparative studies (Hodel *et al.* 2016). The advent of long-read sequencing technologies provides the means to overcome these restrictions. The Telomere-to-Telomere (T2T) Consortium delivered the first gapless human genome assembly, CHM13, correcting inaccuracies in GRCh38 (Nurk *et al.* 2022), and has since expanded to include six non-human primate genomes (Yoo *et al.* 2025). These high-resolution data and the availability of new analysis tools tailored for TRs (Mousavi *et al.* 2021, Dolzhenko *et al.* 2024) open avenues for comparative studies with unparalleled resolution, enabling the discovery of previously undetected TRs, which may help generate new evolutionary hypotheses.

**Figure 1.**
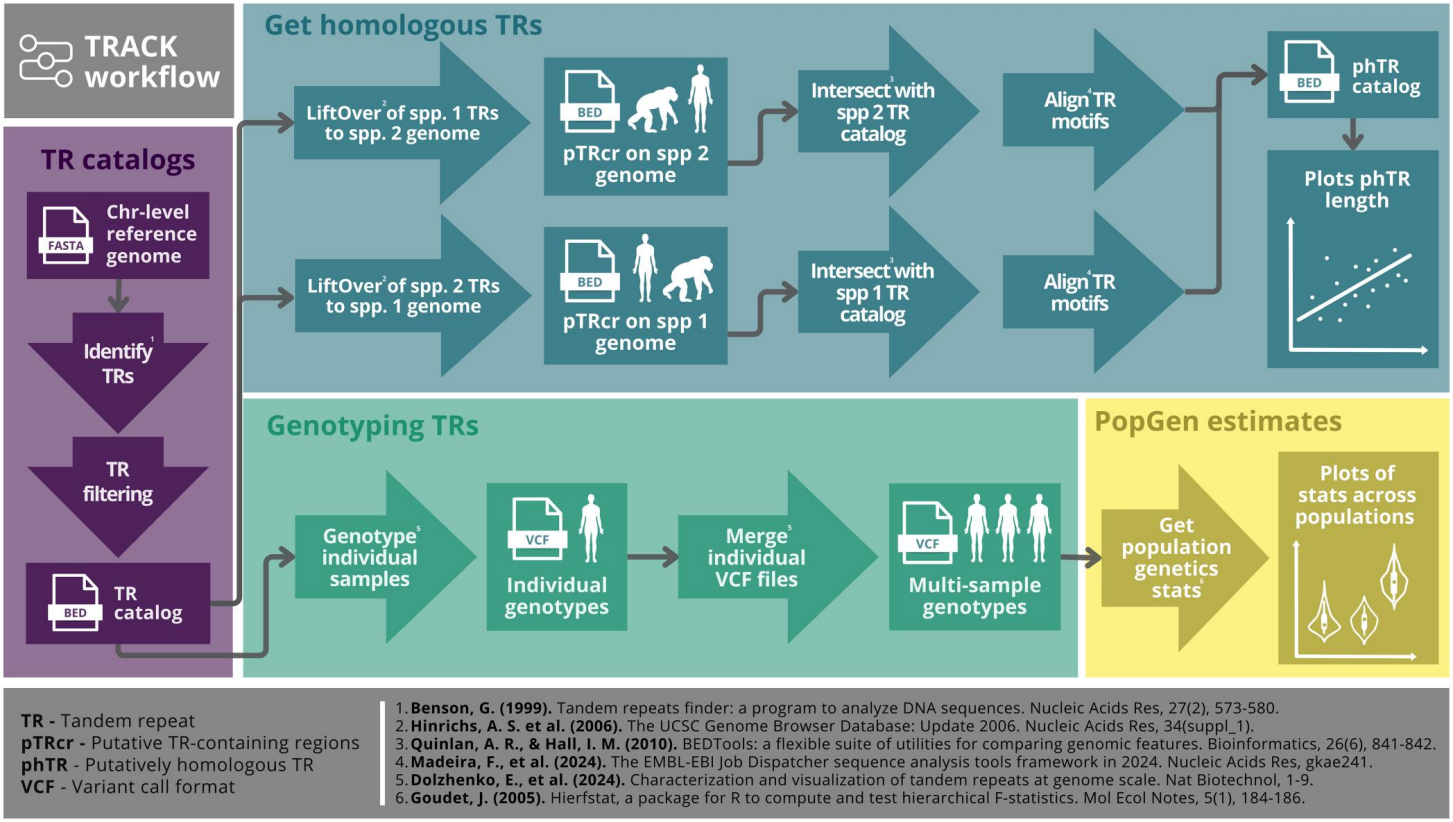
A simplified workflow of the Tandem Repeat Analysis and Comparison Kit (TRACK) features.

A suite of tools has been developed to analyze intraspecific TR variation in long-read data (e.g. Mitsuhashi *et al.* 2019, Chiu *et al.* 2021, Mousavi *et al.* 2021). However, an integrated tool that streamlines the entire process—from generating species-specific TR catalogs to genotyping TRs and assessing homology for interspecific TR variation analysis—is still lacking. To unify the discovery and analysis of shared TR loci across species, we developed the Tandem Repeat Analysis and Comparison Kit (TRACK). This Snakemake workflow integrates TR identification, filtering, homology assessment, and genotyping into a single consolidated tool (Fig. 1).

We built upon well-established TR analysis tools, such as Tandem Repeat Finder (Benson 1999), to identify TRs in chromosome-level genome assemblies, and incorporate Liftover (Hinrichs *et al.* 2006), which converts genomic coordinates between different genome assemblies, to map homologous TR regions between species. Users can define custom thresholds for genomic region overlap and TR motif sequence similarity, allowing fine-tuned putative homologous TR catalog generation. Additionally, TRACK leverages the newly developed Tandem Repeat Genotyping Tool (Dolzhenko *et al.* 2024), tailored for HiFi long-read data, enabling high-resolution TR analysis in population-level datasets. Unlike other long-read genotyping tools (Mitsuhashi *et al.* 2019, Chiu *et al.* 2021), TRGT provides both allele length and sequence composition, allowing the analysis of non-constant variants, i.e. where the fraction of nucleotides within a given TR that matches the consensus motif is <100%. TRACK also enhances usability by providing processed genotype summaries and allele distributions in an accessible tabular format, enabling downstream analyses without requiring direct manipulation of VCF files. This integrated approach improves efficiency and reproducibility, reducing the need for extensive post-processing and custom scripting.

## 2 Pipeline description

### 2.1 Generating TR catalogs

TRACK uses Tandem Repeat Finder (TRF) version 4.09 (Benson 1999) to generate the TR reference catalogs from chromosome-level reference genomes. TRACK default parameters are in Table 1, which results in catalogs with TR minimum length >12 bp. To eliminate redundancy from multiple computations at the same genomic index position or variation in score values, overlapping TRs are initially merged. Subsequently, TRs exceeding 10 Kbp in total length or having a copy number <2.5 are filtered out.

**Table 1.** TRACK default parameters for Tandem Repeat Finder (TRF) run.

Parameter	TRACK default value
Match score	2
Mismatch score	5
Indel score	7
Matching probability	80
Indel probability	10
Minimum alignment score	24
Maximum period size	2000

### 2.2 Identification of putative homologous TRs

utative TR homology is assessed in a pairwise manner. TRACK begins by conducting a LiftOver (Hinrichs *et al.* 2006) analysis using a TR reference catalog from a target genome (tTRc) and a chain file that describes the conversion between genome positions from a target genome assembly to a query genome assembly. Chain files can be obtained directly from the UCSC Genome Browser or be custom-made by the user by performing whole-genome alignment of target and query genomes and subsequent conversion of the alignment file to chain file. The liftover step produces a file of putative TR-containing regions (pTRcr) in the query genome. To reduce bias in homology detection, TRACK performs the LiftOver analysis bidirectionally, with the two genomes of interest serving as both target and query. This yields two lifted bed files for pairwise comparison. The resulting pTRcr file is then intersected with the original TR reference catalog of the query genome used in the comparison, retaining only regions that meet a user-defined overlap threshold. This means that the lifted region and the corresponding TR in the reference catalog must overlap by at least the specified percentage to be kept for further analysis.

**Figure 2.**
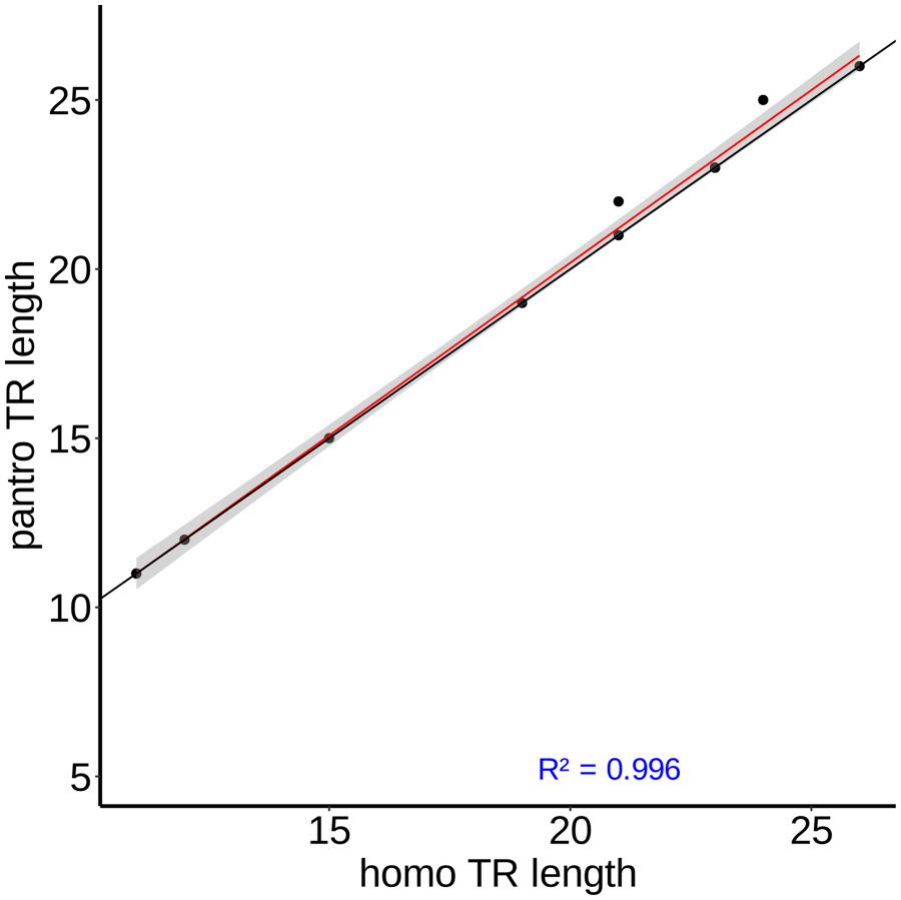
Scatterplot of the total length of 1000 randomly subsetted TRs from the shared TR catalog between human and chimpanzee T2T reference genomes using a threshold of 10% overlap and 95% sequence similarity.

To verify the similarity of these TRs based on sequence composition, TRACK conducts pairwise global alignments between the motifs of the putative homologous TRs from each species using the Needleman-Wunsch algorithm (Madeira *et al.* 2024). Then, TRACK keeps TRs that meet a user-specified threshold for motif sequence similarity. The two resulting files from each pairwise comparison are intersected to produce a final catalog of putatively homologous TRs (phTRs) in bed format. Each row contains the index position, motif and TR lengths, and sequence composition of the homologous pair. TRACK also provides a visualization feature to create a scatterplot comparing the total length of shared TRs between two individuals of different species (Fig. 2).

### 2.3 Genotyping TRs and basic population genetic statistics

**Figure 3.**
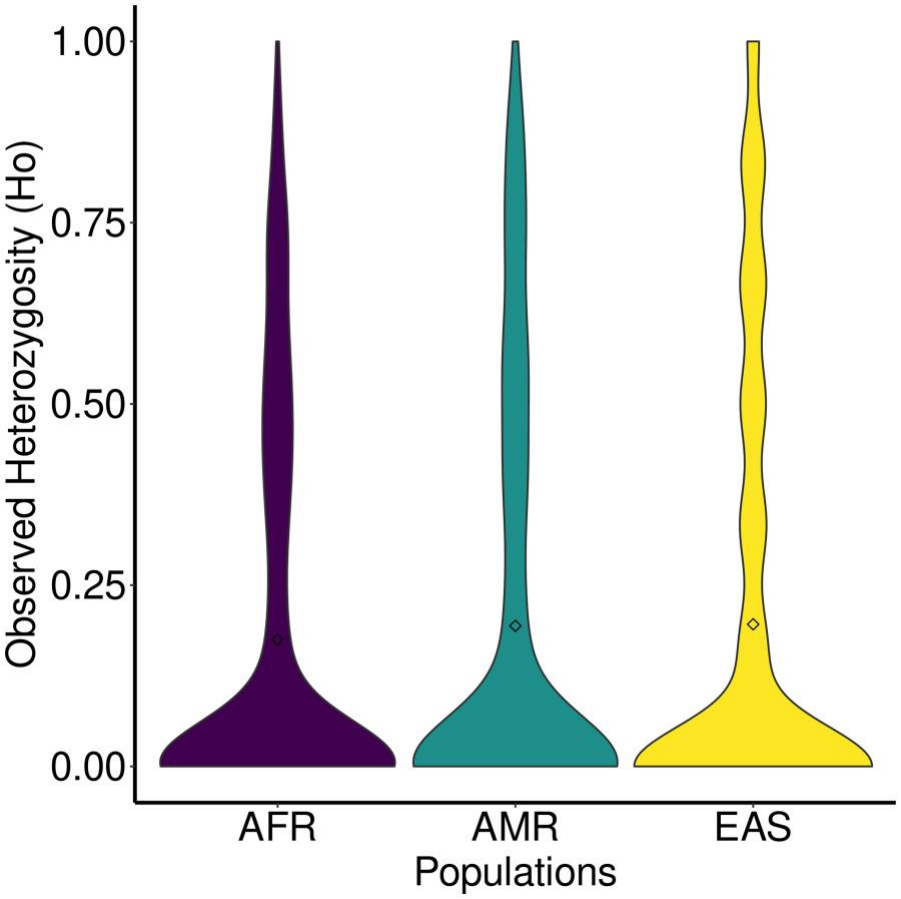
Observed heterozygosity estimates from a random subset of 1000 TR loci from TRACK's human T2T TR catalog genotyped in individuals of the Human Pangenome Reference Consortium (HPRC) (Wang *et al.* 2022). AFR—Africa; AMR—Americas; EAS—Asia.

One of the many uses of TR catalogs is genotyping variants in within-species population datasets. TRACK provides an integrated module for preparing and structuring long-read data to genotype TR variants using the Tandem Repeat Genotyping Tool (TRGT) (Dolzhenko *et al.* 2024). The output is a VCF file containing genotyped TR variants across multiple samples. Additionally, TRACK estimates basic population genetic statistics, such as observed heterozygosity and genetic diversity, and generates violin plots to visualize the results (Fig. 3). TRACK’s genotype functionality also converts the merged VCF into a data frame containing genotypes per sample for each TR. Additionally, TRACK also provides a data frame containing estimates for the number of unique alleles in the VCF and the range of allele lengths per locus.

## 3 Benchmarking

The benchmarking runs were performed using the chimpanzee and human T2T TR catalogs available in the TRACK repository, which contain 3 076 618 (225MB) and 2 756 609 (186MB) repeats, respectively. Bidirectional liftover required ∼254 MB of RAM and 30.32 s of actual CPU time, completing 1m7s of total wall-clock time. Motif sequence alignment had a total runtime of 8 h 20 m, 1.96 h of cumulative CPU time, and 1.9 GB RAM, running with 18 processor threads in parallel. All analyses were performed on a Lenovo Legion personal computer with a 13th-generation Intel Core i7-13700H processor.

## 4 Conclusion

TRACK proposes a novel, streamlined approach to identifying shared TRs between species, simplifying some of the often complex and time-consuming steps of comparative TR analysis. By integrating multiple features from different established tools—from catalog generation and cross-species comparison to population-level genotyping and diversity estimates—TRACK improves the accessibility of TR analyses while increasing reproducibility across analyses. Its flexibility allows users to initiate analyses at different pipeline stages, accommodating diverse research needs. As genomic data continues to expand, tools like TRACK are indispensable for uncovering biologically meaningful insights into TRs, particularly from long-read sequencing data.

## Data Availability

The data underlying this article are available in *caroladam/track* at https://github.com/caroladam/track.
